# Differences in Root Morphologies of Contrasting Wheat (*Triticum aestivum*) Genotypes Are Robust of a Drought Treatment

**DOI:** 10.3390/plants12020275

**Published:** 2023-01-06

**Authors:** Zhuanyun Si, Emmanuel Delhaize, Pieter-Willem Hendriks, Xiaoqing Li

**Affiliations:** 1 Key Laboratory of Crop Water Use and Regulation, Ministry of Agriculture and Rural Affairs/Institute of Farmland Irrigation, Chinese Academy of Agricultural Sciences, Xinxiang 453002, China; 2 CSIRO Agriculture and Food, Canberra, ACT 2601, Australia; 3 Australian Plant Phenomics Facility, Research School of Biology, The Australian National University, Canberra, ACT 2600, Australia; 4 Faculty of Agriculture and Life Sciences, Lincoln University, 85084 Ellesmere Junction Road, Lincoln 7647, New Zealand

**Keywords:** roots, seminal, nodal, lateral, root angle, drought, Spica, Maringa

## Abstract

We aimed to assess the effect of water deprivation on root traits and to establish if the wheat cultivars Spica and Maringa would be useful as parental germplasm for a genetic analysis of root traits. Plants were grown in two markedly different soils under well-watered and water-limited treatments in controlled environment growth cabinets. The drought treatment was imposed as a gradual depletion of water over 28 days as seedlings grew from a defined starting moisture content. The root traits analyzed included length, nodal root number, thickness and nodal root angle. The relative differences in traits between genotypes generally proved to be robust in terms of water treatment and soil type. Maringa had a shallower nodal root angle than Spica, which was driven by the nodal roots. By contrast, the seminal roots of Maringa were found to be similar to or even steeper than those of Spica. We conclude that the differences in root traits between Spica and Maringa were robust to the drought treatment and soil types. Phenotyping on well-watered soil is relevant for identifying traits potentially involved in conferring water use efficiency. Furthermore, Spica and Maringa are suitable parental germplasm for developing populations to determine the genetics of key root traits.

## 1. Introduction

Drought is a major constraint on agricultural production around the world, and with climate change, the severity of drought and its frequency will increase in many regions. Studies have shown that more than 40% of interannual wheat production variability is mainly due to heat waves and drought conditions throughout the world [1]. Under drought, roots are the first organ exposed to the drying soil and the origin of the signals that coordinate the plant’s response [2]. Optimization of the root system is critical for developing crops that are better adapted to a drying climate. 

Wheat has a dual root system consisting of a seminal root system that develops from the embryonic primordia of the seed, forming a primary root with other seminal roots, and nodal roots that develop later from the basal nodes of the main stem and tillers [3,4]. The seed-borne primary and seminal roots are important for early vigor and establish a framework to explore the soil for nutrients and water [5]. Nodal roots emerge from underground shoot nodes and play an important role in the uptake of water and nutrients in later development [3,4]. Lateral roots form on both types of roots within the soil and contribute to the absorption of water and nutrients [6] and can become the main contributor towards the total length of a root system. Seminal roots penetrate soils deep down the profile to enable the extraction of available water at depth late in the growing season [7]. By contrast, nodal roots and their laterals are mainly distributed in shallower soil and maximize water uptake from intermittent, in-season rainfall [8,9]. Ahmed et al. [10] showed that for 2-week-old maize plants, water was mainly taken up by lateral roots and the function of the primary and seminal roots was primarily to transport water to the shoot. By contrast, in more mature maize (5-week-old), water was mainly taken up by crown roots (same as the nodal roots that grow below ground), whereas the seminal roots along with their laterals were minor contributors to water uptake [11]. In young barley plants (14–17 days old), seminal roots contributed more towards water uptake compared to nodal roots due to both a greater length and greater hydraulic conductance [12]. However, as found for maize, nodal roots took up most of the water later in development, indicating the importance of nodal root systems in more mature barley plants [13].

A root system is shaped by the interactions between genetic and environmental components that establish a system with which the plant explores the soil for water and nutrients. An ideotype named ‘steep, cheap, and deep’ to optimize water acquisition was hypothesized to enhance the exploitation of deep soil strata under drought [14]. The benefit of this ideotype on grain yield was demonstrated for deep-rooted durum wheat genotypes compared to shallow-rooted genotypes under rain-fed conditions with a terminal drought [15]. However, under irrigated conditions, the shallow-rooted genotypes yielded more than the deep-rooted genotypes, illustrating the strong genetic-environment interaction that influences the benefit root morphology has on grain yield. Other findings include maize genotypes with reduced numbers of nodal roots [16] or reduced branching density of lateral roots [17] that had improved water acquisition from drying soil. These observations highlight the complexity of what is referred to as drought tolerance or water use efficiency. No single trait can be expected to contribute towards water use efficiency across all environments and across a range of plant species. 

Furthermore, the root system of a plant can be plastic when grown in different environments with differing moisture treatments [18]. Schneider and Lynch [19] suggested that the plasticity of a root system could be better understood as a potential trait for breeding. Indeed, the plasticity of rice roots under drought was identified as a key trait for adaptation to drought [20]. For wheat, the genotype Pavon 76 was better at maintaining yield under moderate drought compared to other genotypes because it produced more roots under drought conditions than in well-watered conditions [8]. 

Although morphological changes to root growth in response to drought stress in wheat have been described [21], it is not clear if root morphologies in wheat are so plastic that selections for root traits created under optimal conditions are relevant to plants grown under drought stress. The wheat cultivars Spica and Maringa were previously found to differ in the sizes of their rhizosheaths due to the length of the root hairs [22]. The development of genetic resources, such as recombinant inbred lines or doubled haploid lines, would be of value to map genetic loci controlling root traits. If Spica and Maringa differed for other root traits in addition to root hair length, it would further enhance the value of a mapping population to identify key genetic loci. While transgressive segregation is possible, if the parental genotypes already differ markedly for traits, then that enhances the likelihood that major genes confer the phenotypes. We hypothesized that the root traits of wheat are so plastic in terms of soil type and water supply that screening for traits would require the use of specific soils with a limited moisture content. To test this hypothesis, we screened Spica and Maringa for a range of root traits and quantified these traits when grown on two contrasting soils under well-watered and drought-stressed conditions. We conclude that the differences in the root traits of Spica and Maringa were robust to the drought treatment and two soil types. Phenotyping on well-watered soil is relevant for identifying traits potentially involved in conferring water use efficiency. Spica and Maringa are suitable parental germplasm for developing populations to determine the genetics of key root traits.

## 2. Materials and Methods

### 2.1. Germplasm

The hexaploid wheat (*T. aestivum*) genotypes Spica and Maringa were previously identified as differing markedly in rhizosheath size and root hair length [22]. Spica is an old Australian cultivar that possesses the wild type *Rht* genes (tall) and has been extensively used in the study of late maturity α-amylase [23]. The Maringa line used in our study is a Brazilian cultivar that had the *Rht-B1b* semi-dwarfing gene introgressed [24]. 

### 2.2. Plant Growth

An initial experiment with potting soil was undertaken to compare the basic root morphologies of Spica and Maringa grown under well-watered conditions. The potting soil was a mixture consisting of sieved (4 mm) potting soil (organic matter and loam) and a 1-g L^−1^ of Aboska fertilizer prepared as previously described [25], except that coarse river sand comprised 30% (*v*/*v*) of the mixture. The final soil pH was 5.7. To ensure a consistent bulk density, a uniform weight (3.6 kg) was added to white, opaque polyvinylchloride (PVC) cylinders of 8.6 cm inner diameter and 50 cm height. The experiment was set up as a randomized complete block design in a growth cabinet (Conviron, Winnipeg, MB, Canada) set with a 16-h-photoperiod (23 °C; 750 µmol m^−2^ s^−1^ light density) and 8-hour darkness (15 °C). The soil water content was maintained daily at 21%, as described below. Plants (four replicates per genotype) were harvested 21, 28, and 35 days after planting. 

In a subsequent experiment, the response of the genotypes to drought was assessed. Spica and Maringa were grown in watered- and drought-treated soils, with five replicates in the potting soil described above and four replicates in field soil. The field soil was collected from the CSIRO Ginninderra Experimental Station (35°10′30″ S; 149˚02′33″ E), sieved through a 4-mm mesh before use and the following nutrients were added to the filed soil as mg kg^−1^: 48 N, 50 P, 183 K, 77 S, 3 Mg, and 84 Ca. The nutrients were added to the dry soil as premixed nutrient solutions. Due to the acidity of the field soil (pH = 4.3), 1.5 g of CaCO_3_ kg^−1^ dry soil was added to increase the pH to 6.0. Pots were prepared with 3.5 kg of field soil added to each PVC cylinder. The two soils were run as separate experiments using randomized complete block designs in a growth cabinet set as described above. The seeds were placed on moistened filter paper in Petri dishes, and the dishes were placed in darkness at 4 °C overnight, with a further 2 days of germination in darkness at 23 °C. Similar-sized seedlings were chosen and planted in the PVC cylinders filled with soil. Soil water content was monitored during the experiment by weighing pots. White plastic beads were used to cover the soil surface to reduce water evaporation. The initial soil water content in all pots was 21% (*w*/*w*) for both soils, as this was the pot capacity for the potting soil and 80% of the field capacity of the field soil. Pots were weighed daily, and for watered treatments, water was added to the top to maintain the soil moisture content near 21%. For the drought treatments, no water was provided until the soil water content had reached 10% for the potting soil and 11% for the field soil, after which the pots were watered daily. The final soil water content for drought treatment was tested in a set of initial experiments that followed the same experimental methods described above to establish whether plants experienced drought stress. The soil moisture content of the drought treatments reduced stomatal conductance for both genotypes and for both soils (Supplementary Figure S1), indicating that plants were being drought-stressed. Plants experiencing the drought treatments received water from the bottom of pots after 25 days of growth to maintain the soil’s water content. Photographs of the plants at harvest time are shown in Supplementary Figure S2a. 

An experiment to determine both the nodal and seminal root angles was undertaken by growing plants in the potting soil that had been treated and packed into cylinders as described above. In this case, three of the cylinders for each genotype were composed of clear Perspex plastic that was covered with aluminum foil during the growth of the plant, while the remaining three cylinders were constructed of opaque PVC (a total of six cylinders per genotype). Prior to harvest, the locations where roots had intercepted the sides of the Perspex cylinders were marked and used to calculate the angles of the roots by simple trigonometry. Using this technique, the average angle of the roots was calculated for each replicate cylinder (Supplementary Figure S2b). After 33 days of growth in a greenhouse set to 23 °C during the day and 15 °C at night, plants were harvested, roots were washed out with tap water, and angles of various root types were measured again with a protractor (Supplementary Figure S2c).

### 2.3. Plant Measurements

At the harvest of experiments that used PVC cylinders, roots were washed free of soil and kept in water at 4 °C until they were measured. The numbers of tillers and leaves were noted, and whole shoots were dried at 65 °C and then weighed. Washed-out roots were scanned using a flat-bed scanner, and images were analyzed with WinRHIZO^TM^ (Regent Instruments Inc., Quebec, QC, Canada). The angles between the vertical and each side of the root crown (outer nodal roots) at the widest section, 1.5 cm down from where shoots emerged out of the root crown, were measured with a protractor. The values were averaged for each plant to generate a single replicate value. In the drought experiment, roots were washed out of the soil cores and sectioned into 10 cm segments along the whole root system, starting from the base. The segments were then scanned as described above. For the experiment that assessed both seminal and nodal root angles, individual roots were identified in the washed-out samples as seminal, coleoptile nodal, first leaf nodal, second leaf nodal, third leaf nodal, and fourth leaf nodal (if present). The angles from the vertical of these individual roots were measured with a protractor at 1.5 cm down in vertical depth. For each plant, the angles of the two narrowest seminal roots were averaged and used as a single value for each replicate plant. Zero degrees denoted a perfectly vertical position, whereas 90 degrees were horizontal, such that shallow roots had large angles and steep roots had small angles. An alternate method for assaying seminal root angles only uses young seedlings grown in field soil. The soil was placed inside a transparent pot, which was then placed inside an opaque black pot, and measurements of seminal root angles followed the method of Richard et al. [26]. Three seedlings of each genotype were planted in an alternating pattern in each of 6 pots (a total of 18 seeds were planted for each genotype). The angle between the pair of first seminal roots developing on either side of the primary seminal root was measured after five days of growth by marking the root angles on the clear pot (Supplementary Figure S2d) and measuring angles as described previously [26]. The angle from the vertical was calculated by dividing by two the angle between a pair of seminal roots. 

The latest fully expanded leaf was chosen for measuring the leaf stomatal conductance with the method described in Li et al. [27] in a set of initial experiments.

### 2.4. Statistical Analyses

The statistical software SPSS 21.0 (IBM, New York, NY, USA) was used to undertake a two-way ANOVA, and the Tukey method was applied for multiple comparisons. Different letters denote significant differences at *p* < 0.05. Data for potting and field soils were analyzed separately by two-way ANOVAs, since the two soil types were grown in two different experiments. For some experiments, Student’s *t*-test was applied to compare genotypes, and in some cases, it was used to compare genotypes grown in a specific soil with a particular treatment in isolation from the total data set. 

## 3. Results

### 3.1. Spica and Maringa Differ in Key Root Traits

**Figure 1:**
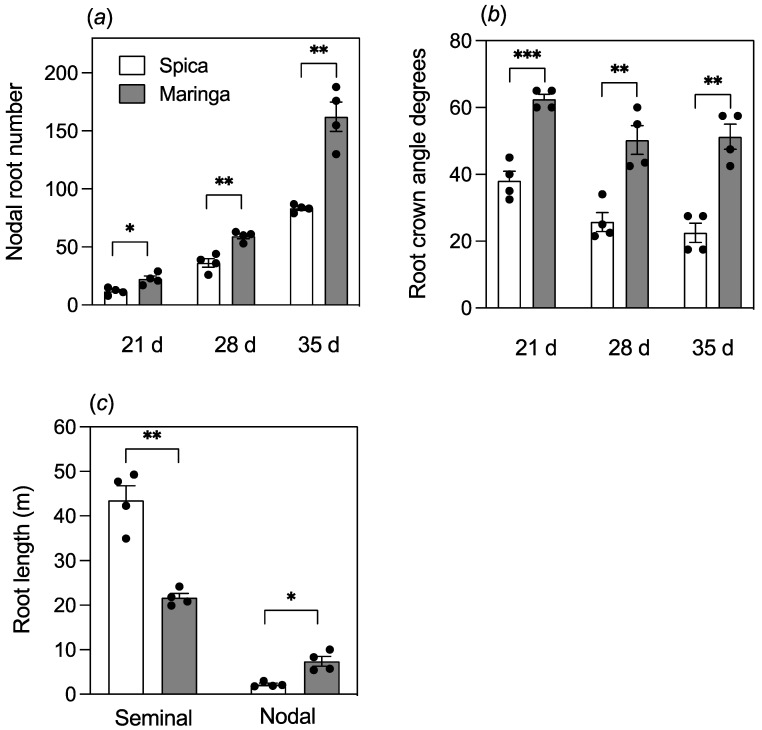
Root morphologies of Spica and Maringa roots differ markedly. Plants were grown in tubes of a well-watered potting soil. Numbers of nodal roots (**a**) and angles of root crowns (**b**) of the two genotypes were measured at each of the three harvests, whereas lengths of seminal and nodal roots were measured at the final harvest (**c**). Individual values are shown as circles, and error bars represent ± SE (standard error) of the mean of four replicates. Data for the genotypes were compared at each harvest with a Student’s *t*-test (* *p* < 0.05; ** *p* < 0.01; *** *p* < 0.001).

An initial experiment over 35 days under well-watered conditions was undertaken to compare basic root traits between Spica and Maringa. Maringa had more nodal roots than Spica with the difference persisting from early growth through to later growth (Figure 1a). Maringa had a wider crown root angle than Spica, and this was evident over the whole growth period (Figure 1b). By the end of the experiment, Spica had longer seminal roots than Maringa but shorter nodal roots (Figure 1c and Supplementary Figure S2e).

Both genotypes followed similar shoot development over 21–35 days, as shown by shoot biomass and tiller numbers (Supplementary Figure S3).

### 3.2. Effect of Drought on Root Traits

**Figure 2:**
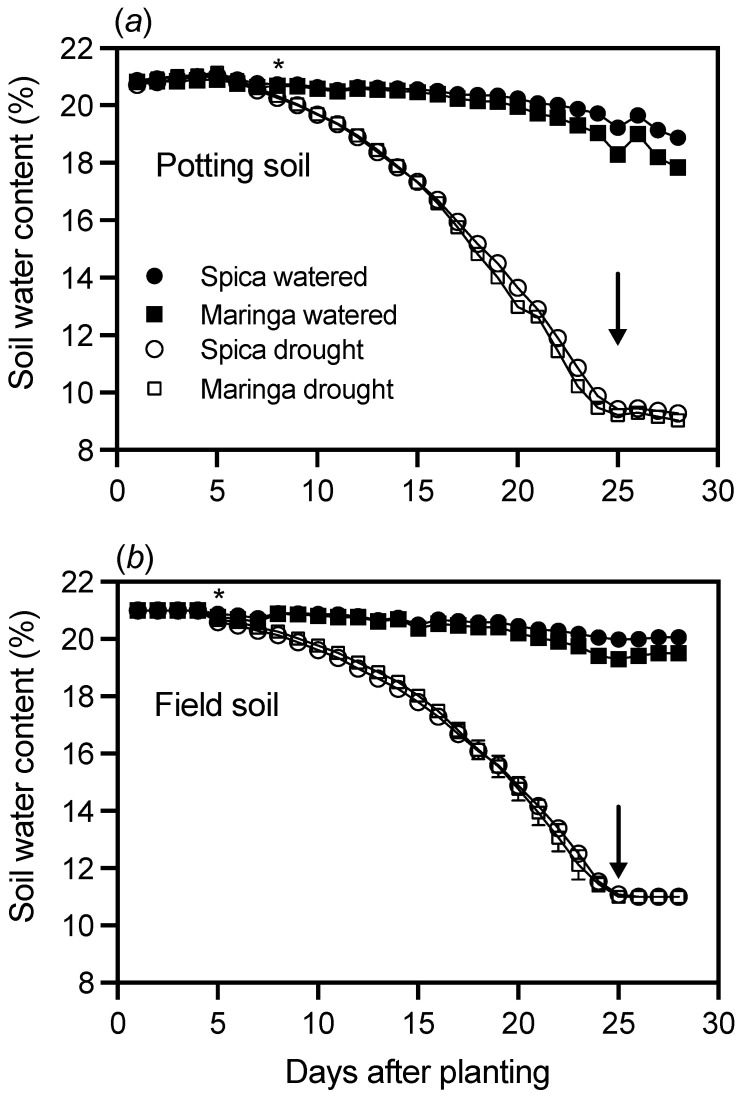
Effect of drought on water content of two soil types in which wheat genotypes of contrasting root morphologies were grown. Watered treatments are denoted by filled symbols and drought treatments by empty symbols, with circles representing pots sown with Spica and squares pots sown with Maringa containing (**a**) potting soil and (**b**) field soil. Where values for Spica and Maringa were sufficiently similar that they overlapped, the symbols representing Spica obscured those representing Maringa. For the drought treatment, after soils were set to their initial water content, they were allowed to deplete until they reached a minimum as denoted by the arrows, after which soil water content was maintained. For watered treatments, the soil water content was maintained daily. Bars denote ± SE of the mean of either four (field soil) or five (potting soil) replicates and are not visible if they are smaller than the symbol. The asterisk denotes the first day where a significant difference (*p* < 0.05) became apparent between watered and droughted pots when data were analyzed with a Student’s *t*-test. Thereafter, the watered and droughted treatments remained significantly different from one another with *p* < 0.001 by day 7 for field soil and by day 8 for potting soil.

Based on the initial experiment, we chose 28 days as a suitable growth period to establish if differences in root traits observed between Spica and Maringa were affected by drought and soil type. The effect of a drought treatment was quantified by growing plants in tubes that were either watered to weight daily or allowed to dry after the soils had started with the same initial moisture content as the well-watered controls. The daily water content of the potting and field soils both declined for the drought treatment, whereas water content was effectively maintained in both soils for the watered treatments (Figure 2). Within each treatment, the soil moisture content was remarkably similar for the two genotypes regardless of the soil type (Figure 2).

**Figure 3:**
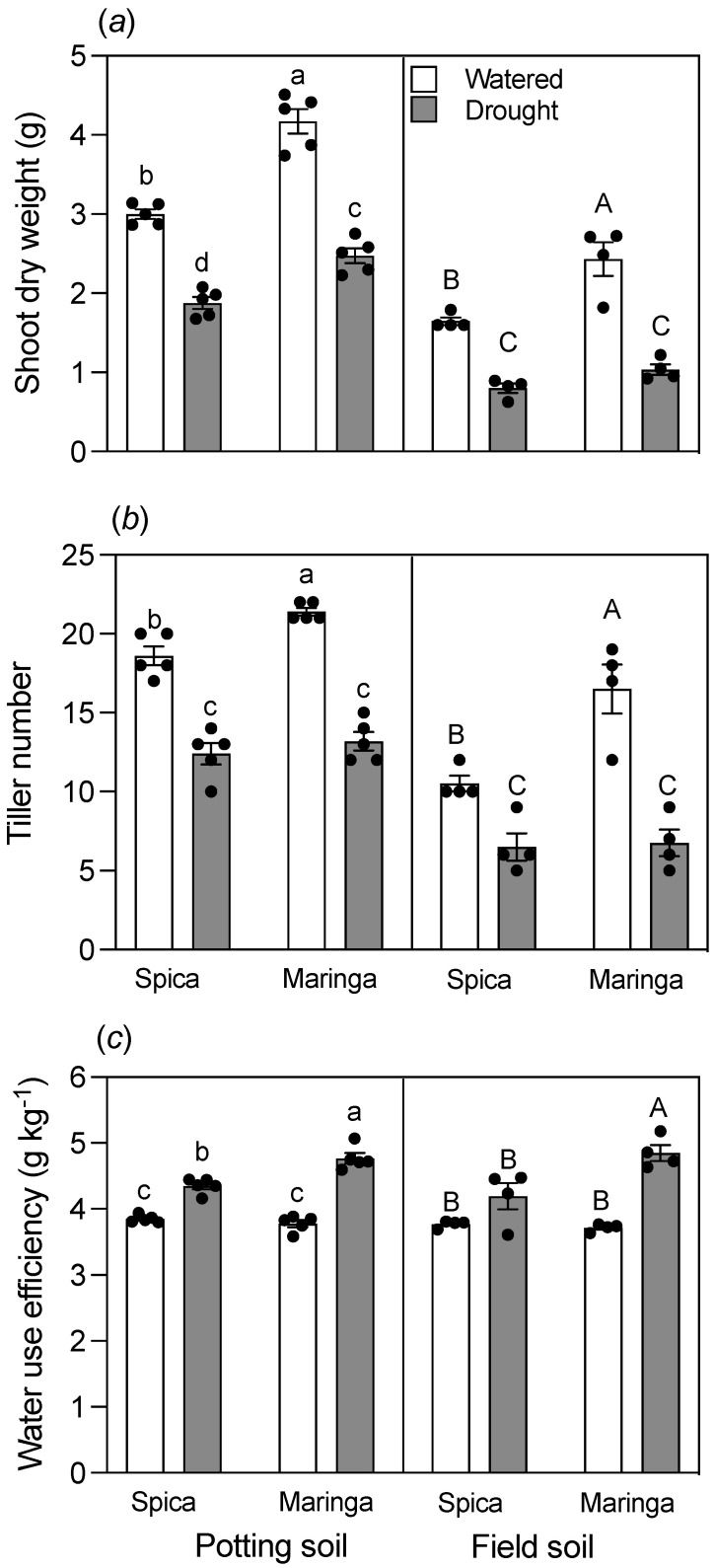
Effect of drought on shoot biomass, tiller number, and water use efficiency. Shoot dry weight (**a**), tiller number (**b**), and water use efficiency (**c**) of genotypes grown for 28 days in well-watered (empty bars) and drought-treated (filled bars) potting and field soils. Water use efficiency is defined as the amount of shoot biomass generated per unit of water taken up. Individual values are shown as circles, and error bars represent ± SE of the mean for either five replicates (potting soil) or four replicates (field soil). Data for potting and field soils were analyzed separately by two-way ANOVAs. Different lowercase letters reflect significant differences between treatments as determined by the Tukey test at *p* < 0.05 for potting soil, whereas different uppercase letters reflect significant differences between treatments at *p* < 0.05 for field soil. The interaction between water and cultivar factors for potting soil was (**a**) significant at *p* < 0.05 for shoot dry weight, (**b**) not significant for tiller number, and (**c**) not significant for water use efficiency. The interaction between water and cultivar factors for field soil was (**a**) significant at *p* < 0.05 for shoot dry weight, (**b**) significant at *p* < 0.05 for tiller number, and (**c**) significant at *p* < 0.05 for water use efficiency.

Maringa produced more shoot biomass and tillers than Spica for the watered treatment of both the potting and field soils (Figure 3a,b). Drought treatment reduced shoot biomass and number of tillers for both genotypes grown in both soils compared to the watered treatments. Water use efficiency, expressed as the amount of shoot biomass produced per unit of water taken up, was the same for both genotypes when grown under watered conditions in both soils (Figure 3c). However, when droughted, Maringa was more water efficient than Spica regardless of the soil type.

**Figure 4:**
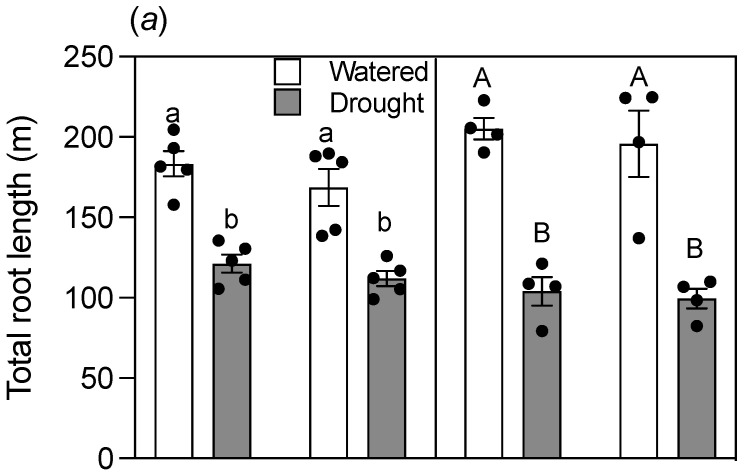

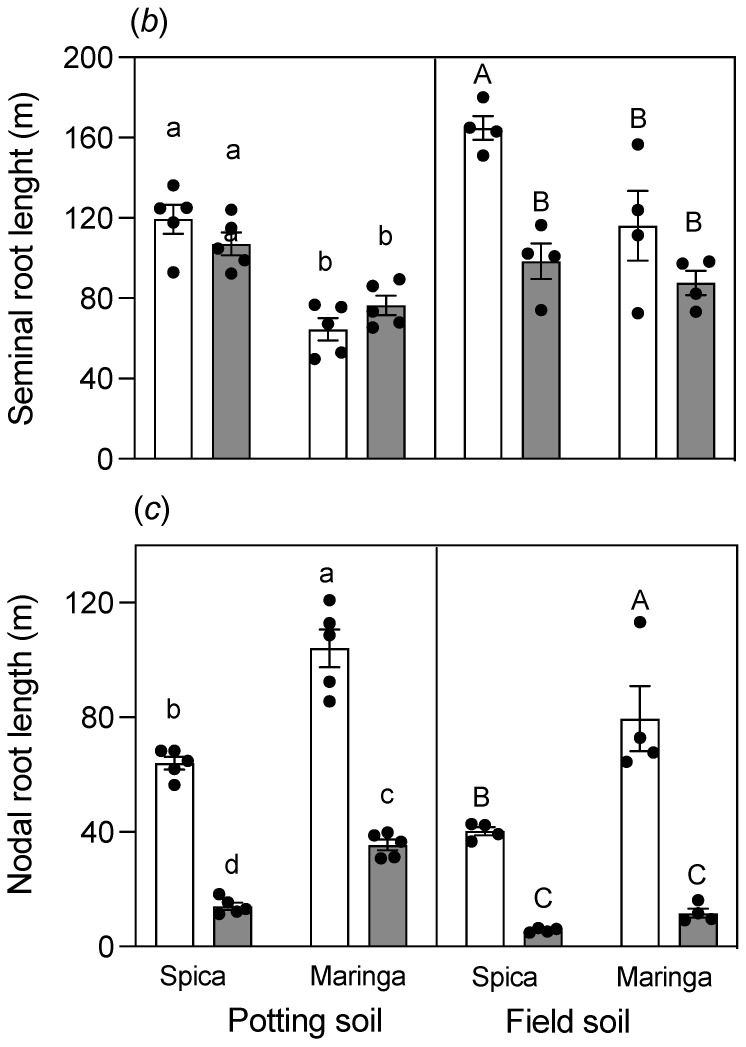
Total root lengths of Spica and Maringa were similar, but the relative proportions contributed by seminal and nodal roots differed. Total root length (**a**), seminal root length (**b**), and total nodal root length (**c**) of the two genotypes in watered (empty bars) and drought (filled bars) treatments after 28 d of growth. Individual values are shown as circles, and error bars represent ± SE of the mean for either five replicates (potting soil) or four replicates (field soil). Data for potting and field soils were analyzed separately by two-way ANOVAs. Different lowercase letters reflect significant differences between treatments at *p* < 0.05 for potting soil, whereas different uppercase letters reflect significant differences between treatments as determined with the Tukey test at *p* < 0.05 for field soil. Analysis of nodal root length of Spica and Maringa under drought in the field soil by Student’s *t*-test in isolation of other data showed a significant difference (*p* = 0.03), whereas the two-way ANOVA did not show significance at *p* < 0.05. The interaction between water and cultivar factors for potting soil was (**a**) not significant for total root length, (**b**) not significant for seminal root length, and (**c**) significant at *p* < 0.05 for nodal root length. The interaction between water and cultivar factors for field soil was (**a**) not significant for total root length, (**b**) not significant for seminal root length, and (**c**) significant at *p* < 0.05 for nodal root length.

The drought treatment reduced total root length (Figure 4a) in both genotypes. Total root lengths of Maringa and Spica were equal in the watered treatment and remained similar to one another under drought despite a large reduction in root length due to the treatment (Figure 4a). Although the genotypes had similar total root lengths, the proportion of length contributed by seminal and nodal roots differed. Spica had longer seminal roots than Maringa for both soils under watered conditions (Figure 4b, Supplementary Figure S2e). Plants grown in the droughted potting soil maintained this difference, whereas the droughted genotypes grown in the field soil did not differ from one another (Figure 4b). Even though drought stress markedly decreased total nodal root length, Maringa maintained longer nodal roots than Spica for both soils under both treatments (Figure 4c). Root weights generally followed similar patterns to root lengths, except for seminal roots grown in field soil (Supplementary Figure S4). The smaller biomass of Spica roots grown in field soil despite similar or longer roots suggested the roots were thinner and had a greater specific length than Maringa. Both of these traits were explored in further analyses described below. Analysis of root to shoot ratios showed only minor or no differences between genotypes despite a large increase in the ratio for both genotypes grown on field soil (Supplementary Figure S5).

Spica had thinner seminal roots than Maringa in both treatments and both soils, whereas the nodal roots of Spica, although thinner under well-watered conditions, did not differ from Maringa under drought (Supplementary Figure S6). Drought stress significantly increased root diameters of both Spica and Maringa across the two soil types. Drought caused a consistent reduction in specific root lengths of seminal and nodal roots for both genotypes, with Spica generally maintaining a larger specific root length than Maringa across both soils in the watered treatment that was not apparent on droughting (Supplementary Figure S7). 

**Figure 5:**
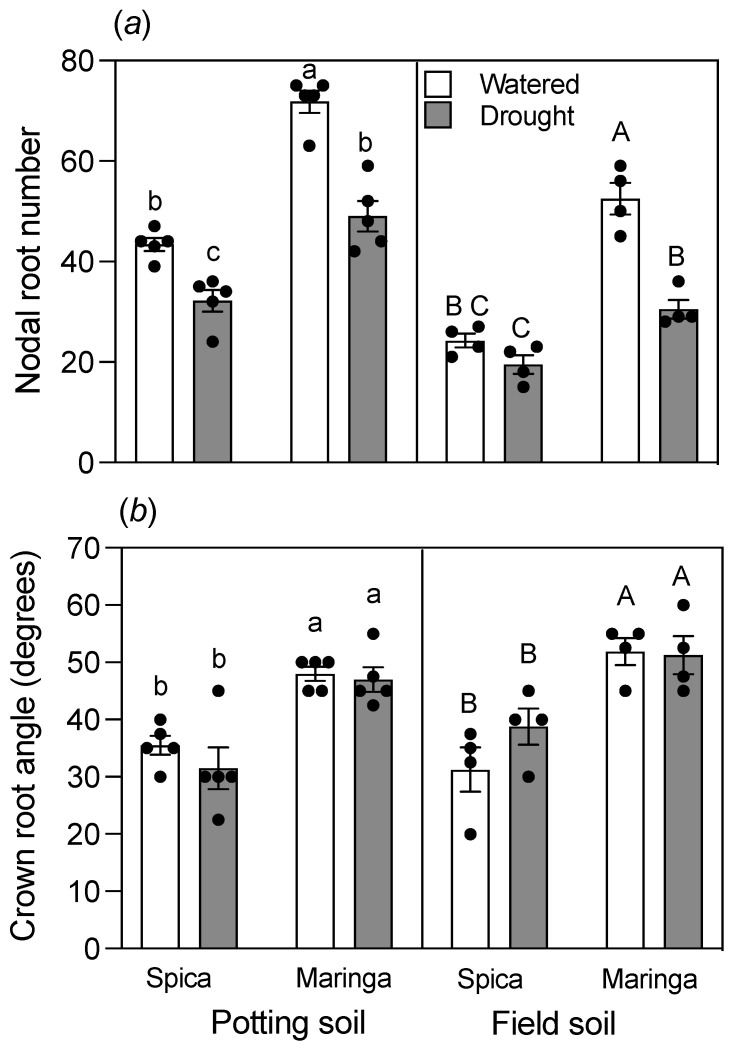
Differences in numbers of nodal roots and crown root angles between Spica and Maringa are robust to soil type and water treatments. Numbers of nodal roots (**a**) and angles of root crowns (**b**) of the two genotypes in watered (empty bars) and drought (filled bars) treatments after 28 d of growth. Individual values are shown as circles, and error bars represent ± SE of the mean for either five replicates (potting soil) or four replicates (field soil). Data for potting and field soils were analyzed separately by two-way ANOVAs. Different lowercase letters reflect significant differences between treatments as determined with the Tukey test at *p* < 0.05 for potting soil, whereas different uppercase letters reflect significant differences between treatments at *p* < 0.05 for field soil. The interaction between water and cultivar factors for potting soil was (**a**) significant at *p* < 0.05 for nodal root number and (**b**) not significant for crown root angle. The interaction between water and cultivar factors for field soil was (**a**) significant at *p* < 0.05 for nodal root number and (**b**) not significant for crown root angle.

Both Spica and Maringa germinated five seminal roots in watered and drought-treated soils. By contrast, drought stress generally decreased the number of nodal roots for both genotypes (Figure 5a). Although the absolute numbers of nodal roots differed between soils, the relative differences in numbers between genotypes were maintained in the drought treatment, with Maringa having more nodal roots than Spica in both soils. Spica consistently had a narrower crown root angle than Maringa in both soils, regardless of the water treatment (Figure 5b). Indeed, the root angle was remarkably robust to both soil type and watering regime. Despite the difference in crown root angle, root densities down the soil profile were similar for both genotypes grown on the same soil and with the same water treatment (Supplementary Figure S8).

### 3.3. Nodal and Seminal Root Angles

**Figure 6:**
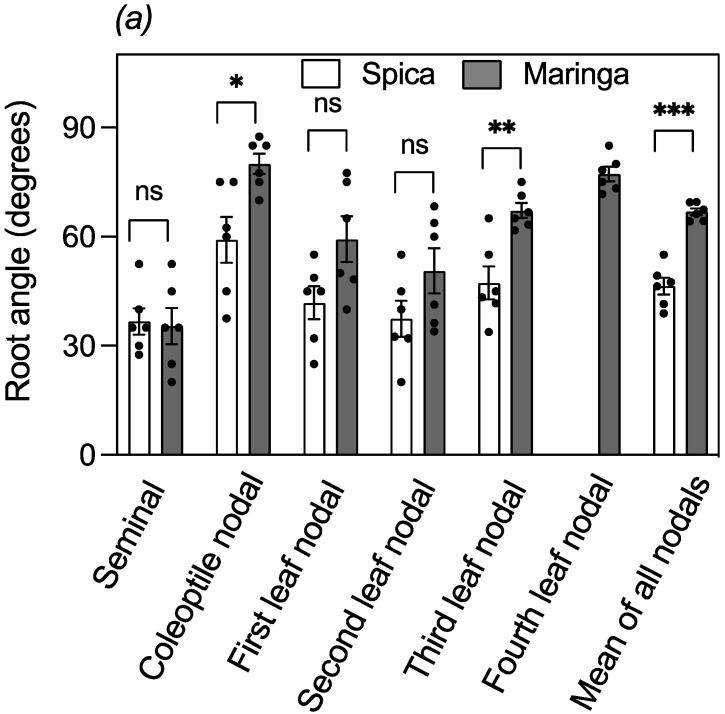

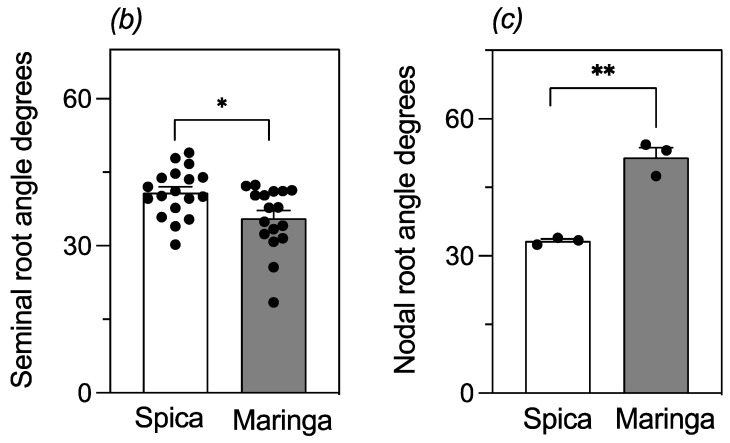
Nodal and seminal root angles of Maringa and Spica. Plants were grown in potting soil for 33 days in tubes after which roots were washed out and root angles for the various classes of roots were measured (**a**; n = 6). Seminal root angles of five-day old seedlings were determined by the clear-pot method as described by Richard et al. [26]. Angles were measured for the first of the seminal roots developing either side of the primary seminal root (n = 18 for Spica and n = 17 for Maringa) (**b**). Nodal root angles after 33 days of growth were determined by measuring the interception of roots against the sides of clear tubes and calculating the angles by simple trigonometry (**c**; n = 3). Individual values are shown as circles with calculated data shown as the mean ± SE, and Maringa was compared to Spica for the various root types using a Student’s *t*-test (* *p* < 0.05; ** *p* < 0.01; *** *p* < 0.001).

To further establish the robust nature of large crown root angles in Maringa and to explore the root angles in different whorls of nodal roots and seminal roots, plants were grown in a greenhouse for 33 days under more varied conditions than experienced in the cabinet. Figure 6a shows Maringa had larger nodal root angles than Spica when data for all nodal root types were averaged. The angles of individual nodal root types were generally larger for Maringa than Spica. By contrast, seminal root angles did not differ between the genotypes (Figure 6a). 

Seminal roots are thinner than nodal roots and are less likely to maintain their shape when washed to remove soil. It was possible that Maringa’s seminal roots had partially collapsed to obscure a larger angle. To avoid this potential problem, the seminal root angle was assessed in situ using a previously described method with young seedlings growing in clear pots [26]. However, the assay showed Maringa had even smaller seminal root angles than Spica, indicative of steeper roots (Figure 6b). Similarly, to verify the nodal root data, we grew a subset of plants in clear tubes covered with aluminum foil during growth. The clear tubes enabled roots to be marked when they intercepted the side of the tube, and the angles they subtended could be calculated. Figure 6c shows that this assay was consistent with data obtained from washed-out roots, confirming that the nodal roots of Maringa had a larger angle than those of Spica.

## 4. Summary

Table 1 summarizes the various traits of Maringa and Spica contributing to root morphology and the effects of watering regime and soil type. Within a row, Spica and Maringa are largely consistent, such that the same relative ranking was maintained across soil type and watering regime. There are exceptions, such as nodal root diameter, which did not show a difference under drought for both soils, whereas Spica maintained a smaller nodal root diameter under watered conditions for both soils. 

## 5. Discussion

The comparison of Spica and Maringa confirmed that these wheat genotypes had contrasting root systems. Although the root traits showed a degree of plasticity, the relative differences between Spica and Maringa were largely robust to drought and soil type. For instance, although nodal root length and number were reduced by the drought treatment for both genotypes, Maringa still maintained longer and more nodal roots than Spica (Figure 4c and Figure 5a). In our study, we conclude that the hypothesis asserting that the plasticity of root systems when screening for root traits relevant to dry soils requires specific soils with limited moisture content is not correct. Spica had longer and thinner seminal roots than Maringa, whereas Maringa had more and longer nodal roots than Spica (Figure 1, Figure 4, and Figure S6). Analysis of the angles of the various root types showed that the larger crown root angles along with an analysis of individual nodal root types for Maringa, were indicative of shallower roots (Figure 6). By contrast, Maringa had steeper seminal roots, as determined by a method that measured roots in situ. These findings show that the different root types of wheat can differ in their gravitropic responses. In some species, differing gravitropic responses by different root types can be extreme, as found in mangroves, where pneumatophores grow upwards against gravity while other roots grow horizontally or downwards [28]. Although not as extreme, the different gravitropic responses of nodal and seminal roots in wheat could be exploited to develop root systems suited to varied environments, as discussed below. Understanding the biochemical and molecular bases for the differing gravity responses of different root types would be an interesting topic to pursue, but is beyond the scope of the current research. 

The strongly contrasting root systems of Spica and Maringa indicate that these genotypes are useful parental germplasm for genetic studies in developing root systems suited to drought conditions. The type of drought can vary considerably, with some regions experiencing dry conditions where water is scarce at the soil surface but is available at depth, and under these conditions, deep-rooted genotypes with narrow root angles are likely to perform best [14,29]. By contrast, shallow-rooted genotypes with a large crown root angle would be better suited to a region that experiences intermittent rainfall or irrigation over a growing season. For example, durum wheat (*T. turgidum*) genotypes with deep roots yielded best under drought conditions, whereas shallow-rooted genotypes had an advantage under well-watered conditions [15]. Sadras and Rodriguez [30] studied rainfall patterns in eastern Australia to conclude that deep-rooted wheat genotypes would be of greater value in northern locations where there are substantial pools of stored water at depth, whereas the production of superficial or shallow roots where intermittent rainfall predominates could be of greater value in southern locations. Large root size is thought to be beneficial to water use efficiency in wheat, as described for the wheat genotype Pavon 76 [8]. This suggests that long roots with a large surface area are key morphological traits associated with drought tolerance for improved productivity in mature plants. However, in environments where crops are reliant on stored soil water at depth, a vigorous root system increases the risk of depleting soil water before completion of grain filling [31]. In support of this notion, a study of two wheat cultivars that differed in root sizes, the cultivar with the small root system conserved water during growth such that it coped with a terminal drought more effectively than the large-rooted cultivar, which depleted soil water more rapidly, resulting in restricted grain filling [32]. Furthermore, climate change resulting in elevated CO_2_ concentrations could exacerbate the effects of vigorous wheat genotypes depleting soil water before grain filling is complete [33]. This discussion illustrates the point that traits that may be beneficial for water use efficiency of plants grown in one environment could be detrimental to water use efficiency in another environment. 

Plants grown in pot experiments do not replicate a crop grown in the field, and a range of factors need to be considered for improving water use by crops grown in the field [34]. Nevertheless, pot experiments can be run under controlled conditions and increase the throughput of genotypes analyzed compared to the field experiments, where each season can vary. An example of a high-throughput assay for seminal root angle illustrates the value of such a method for analyzing large numbers of genotypes [26]. Although this type of assay targets a single root trait, the seminal root angle of young seedlings is associated with root depth and is based on previous data showing that deep roots in some environments improve water uptake [7]. The screen described in the current paper is not as high throughput as the one described by Richard et al. [26], but it included analyses of root traits that are not apparent in young seedlings. For example, the rapid screening was undertaken over a short period before nodal roots had appeared. Nodal roots predominate late in a growing season, constituting the majority of the root system of a wheat plant, and selection for attributes specific to nodal roots may be of value to water use. Whatever screening method is adopted, the plants selected with the desirable trait ultimately need to be assessed in the field with suitable control genotypes to determine the benefits of the trait. 

Although roots are key for taking up water and an obvious target for improving water use efficiency, the effect of shoots on water use should not be ignored. An intriguing finding from our current work is that Maringa was more water efficient than Spica (Figure 3), and this is unlikely to have been a consequence of the differences in root morphology as both genotypes were able to deplete similar amounts of soil moisture despite having different shoot sizes. Pot trials typically restrict root growth unless very large pots are used, and it is unlikely that an attribute, such as narrow and deeper roots would have affected water use efficiency in our experiments. Although our experiments avoided the common problems described by Passioura [35] in running pot trials, the size of the pots would have still restricted root distribution of the plants grown for 28 days. The final distribution of roots down the soil profile of the droughted treatment did not differ markedly between Spica and Maringa, indicating that Maringa did not access a pool of moisture that was less available to Spica due to differences in root morphology (Supplementary Figure S8). However, it is still possible that root morphology is key for water use efficiency in the field and that assessment of the genotypes in the field under varied water supplies would overcome the limitations imposed by pots. 

Root morphology is not only important in determining how effectively plants take up water, but also influences the efficiency of how well mineral nutrients are taken up. The two major nutrients, N and P, serve as contrasts for comparing the types of traits that would improve the uptake of each nutrient. Although a large root system would generally be considered the most effective trait for the uptake of both N and P, the most effective morphology for a root system to enable efficient uptake of each nutrient differs. P is primarily taken up by plants as phosphate, and in soil, phosphate is generally poorly mobile, such that it is often stratified down the profile with the majority of the phosphate found in surface soil [36]. Bean genotypes that explored the surface soil were found to be more P-uptake efficient than deep-rooted genotypes [37]. By contrast, a major form of soil N that is accessible to plants is nitrate and this highly mobile ion is readily leached by water moving down a soil profile so that deep roots are better suited at capturing the nitrate [14]. These contrasting nutrients would suggest that designing an effective root system is a compromise and that an efficient root system for both nutrients is not possible. However, the monocotyledonous root system is comprised of seminal roots and the later developing nodal roots. Here, we show that Maringa had shallow nodal roots compared to Spica, yet its seminal roots were similar to or even steeper than those of Spica. Showing that these two major root types can differ in their properties indicates it is possible to have a root system that combines deep seminal roots for effective uptake of deep water or nitrate with less gravitropic nodal roots that are able to explore the surface soil for phosphate or water uptake from intermittent rainfall. 

In view of their contrasting root systems, we crossed Spica and Maringa to generate a population of recombinant inbred lines. In the current manuscript, we characterized the parental lines in detail to assess the degree of root plasticity when plants were grown under-watered and drought conditions in two soils that differed markedly in their properties. Some of the root traits responded to drought as expected, such as decreases in nodal root length and nodal root numbers. Where root traits varied due to the drought treatment, the relative differences were generally maintained. Table 1 shows the overall consistency of the relative root morphologies of plants grown in the contrasting soils under-watered and drought treatments. There were no instances where scoring of a trait was found to be reversed under drought compared to the watered treatment. These findings have implications for experiments aimed at screening wheat germplasm for root traits that could improve water use efficiency. For instance, wheat germplasm could be screened under watered conditions to avoid complications due to imposing a drought treatment. Typically, rapid screens are first undertaken in seedlings or young plants, and then a subset of promising germplasm is assessed where plants are taken to maturity under drought and ultimately in the field to establish if a particular trait has value in improving the efficiency of water use and nutrient uptake. In our work, although the roots showed a degree of plasticity when there is a drought, the relative differences between the genotypes were largely maintained. This was particularly true of root angle, which proved remarkably robust to soil type under both watered and drought treatments (Figure 5b). 

## 6. Conclusions

The two genotypes characterized in this paper have traits, such as deep roots that could be beneficial to environments where reliance on stored water predominates, as well as traits, such as shallow roots that could be beneficial where intermittent rainfall is common. Furthermore, we show these traits need not be a compromise, as the root system of wheat can have shallow nodal roots coupled with steep seminal roots. The experimental methods described in this paper will be useful for screening a population of recombinant inbred lines that have been developed using Spica and Maringa as parental genotypes and can also be applied to other genotypes and species where root traits are to be measured. Traits, such as crown root angle, number of nodal roots, and seminal root diameter, proved robust to soil type and drought conditions. These findings show that the screening method with an adequate water supply could be applied to screen recombinant inbred lines to determine the genetics of selected traits, for example, root traits that improve water use efficiency.

## Figures and Tables

**Table 1:** Relative properties of root traits for Spica (S) and Maringa (M) grown in two soil types under watered and drought conditions.

Traits	Watered				Drought			
	Potting Soil		Field Soil		Potting Soil		Field Soil	
	S	M	S	M	S	M	S	M
Total root length	=		=		=		=	
Seminal root length	>		>		>		=	
Nodal root length	<		<		<		<	
Nodal root number	<		<		<		<	
Root angle	<		<		<		<	
Seminal root diam.	<		<		<		<	
Nodal root diam.	<		<		=		=	

“=” denotes an equal phenotype; “>” indicates a relatively higher value for Spica; “<” indicates a relatively lower value for Spica.

## Data Availability

The data that support this study are available in the article and accompanying online supplementary material. The raw data in this study are available upon request from the corresponding author.

## Supplementary Materials

The following supporting information can be downloaded at: https://www.mdpi.com/article/10.3390/plants12020275/s1, Figure S1: Stomatal conductance; Figure S2: Photographs of shoots and roots; Figure S3: Shoot development of Spica and Maringa; Figure S4: Root weights of Spica and Maringa; Figure S5: Root to shoot ratios; Figure S6: Average root diameters; Figure S7: Specific root length; Figure S8: Root density distribution down soil profiles.
