# Supplementary material for: Differences in Root Morphologies of Contrasting Wheat (Triticum aestivum) Genotypes Are Robust of a Drought Treatment
Source: Plants (Basel). 2023 Jan 6;12(2):275. doi: 10.3390/plants12020275 (PMC9863919; doi:10.3390/plants12020275)
Supplement: Supplementary file 1 [file plants-12-00275-s001.zip › plants-2023269-supplementary.pdf]

Supplementary Figures

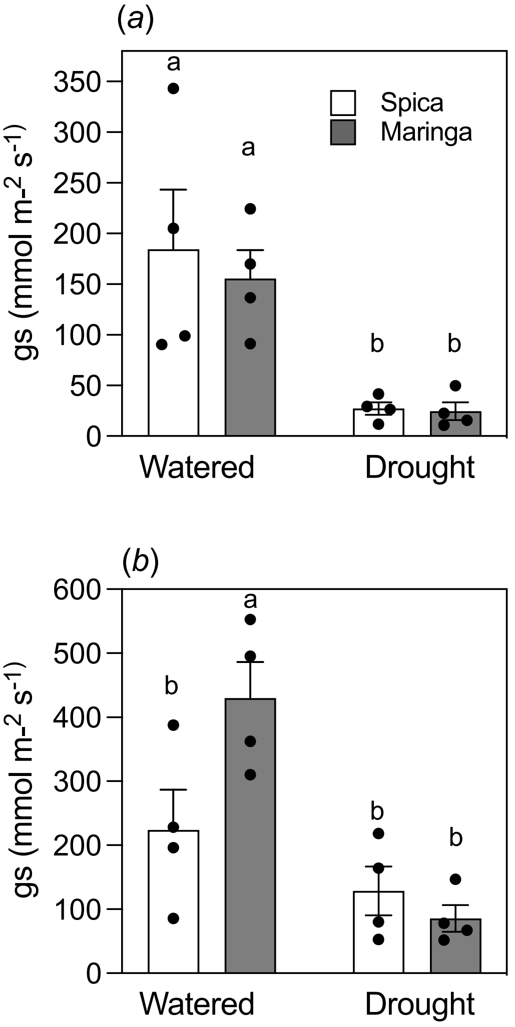

Figure S1 Stomatal conductance of the latest fully expanded leaf of plants grown in (a) potting soil, and (b) field soil. Measurements were taken at 28 days after sowing. For drought treated pots, the soil water content in potting soil was 9.3% for Spica and 9.0% for Maringa, and in field soil it was 11.0 % for both genotypes. The soil water content of 10% for potting soil and 11% for field soil were set as the threshold for drought treatment in subsequent experiments. Individual values are shown as circles and error bars represent ± SE (standard error) of the mean of four replicates. Data for were analysed by two-way ANOVA. Different lowercase letters reflect significant differences between treatments as determined with the Tukey test at $P < 0.05$. The

interaction between water and cultivar factors for potting soil was (a) not significant whereas the interaction between water and cultivar factors for field soil was (b) significant at $P < 0.05$ for stomatal conductance.

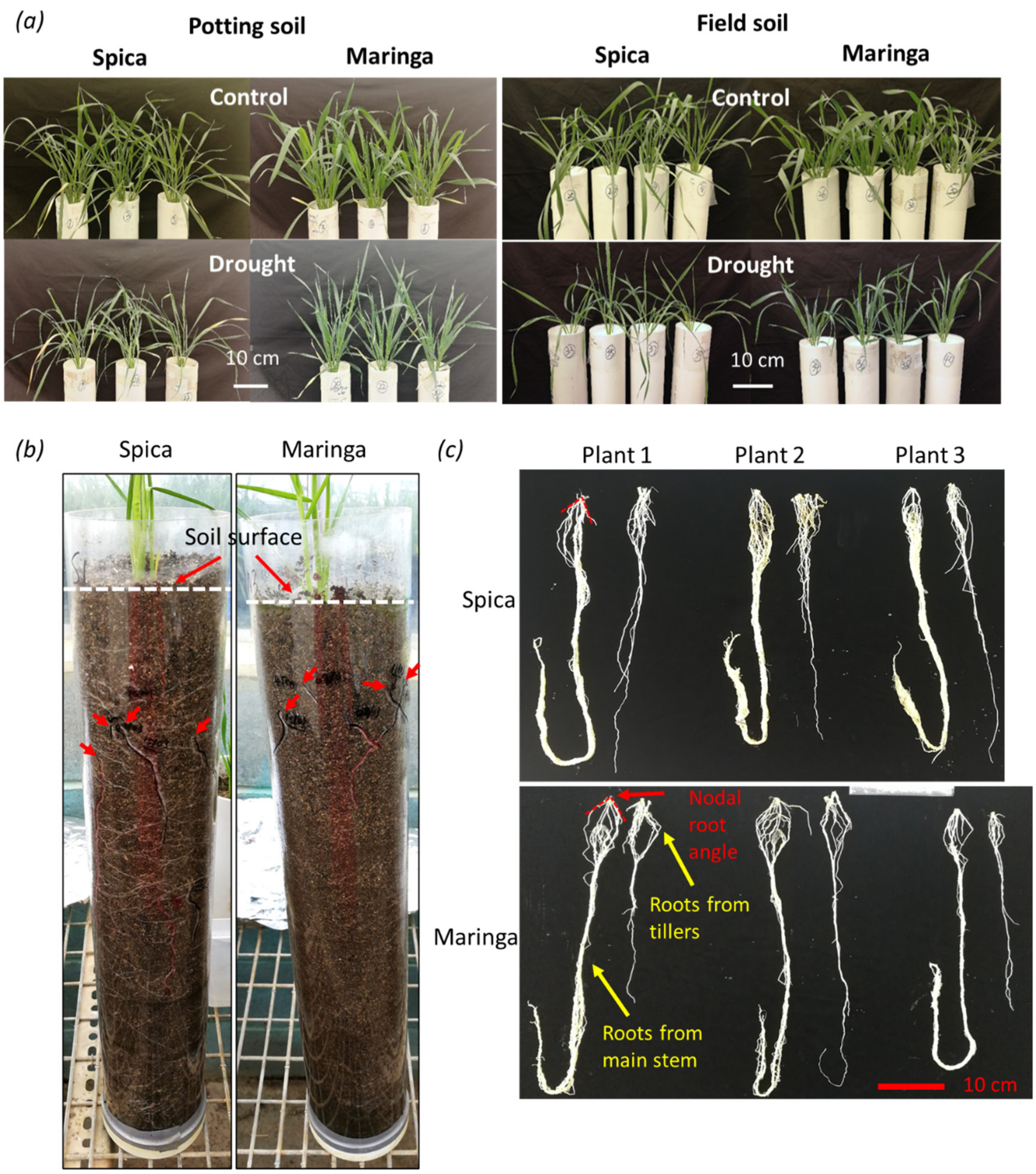

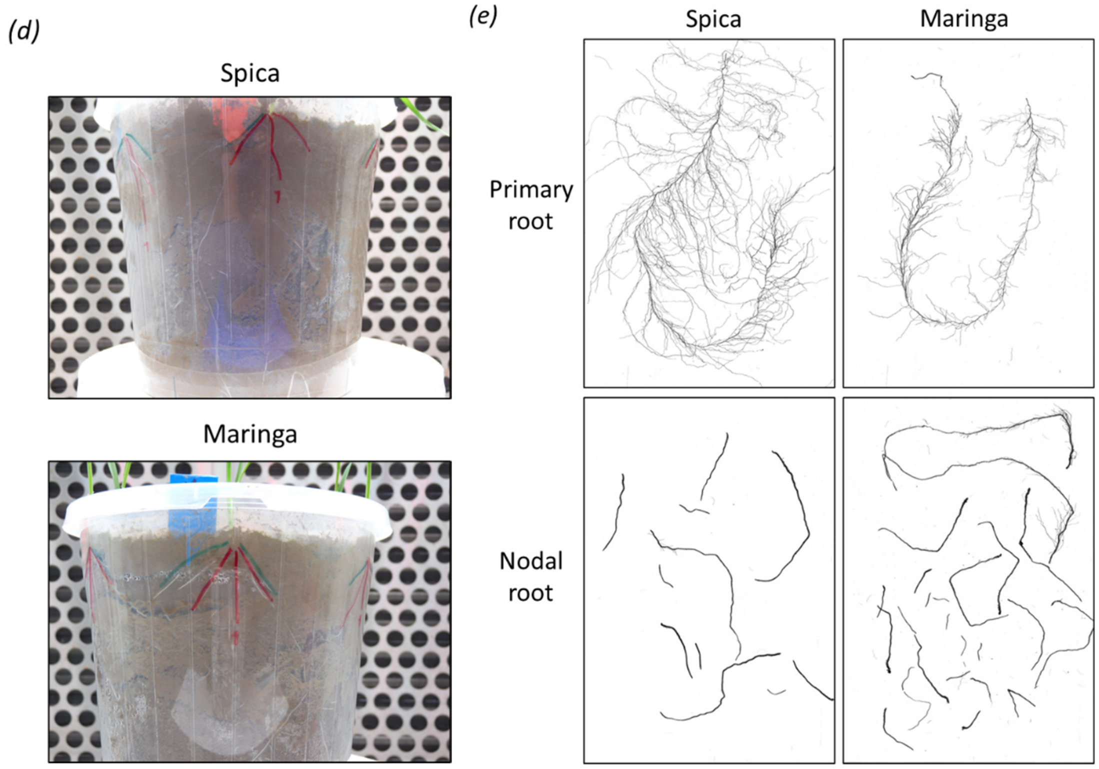

Figure S2 Photographs as examples illustrating the experimental methods of (a) plants in the end of the drought experiment with both potting soil and field soil; (b) nodal roots marked on the surface of the transparent pots at 23 days after sowing. Red arrows indicate nodal roots where they first touched the pot surface; (c) washed roots separated from shoots after harvest (33 days after sowing); (d) plants grown in transparent pots with field soil for seminal root angle measurement; (e) scanned primary and leaf nodal roots (21 days after sowing).

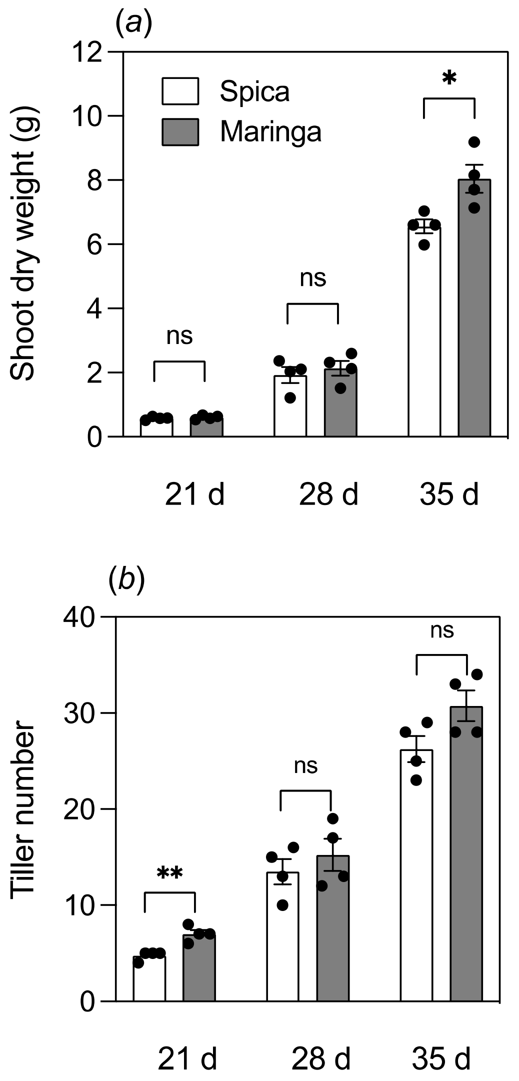

Figure S3. Shoot development of Spica and Maringa over thirty-five days. Plants were grown in tubes of a well-watered potting soil. Shoot dry weight (a) and tiller numbers (b) of the two genotypes were measured at each of three harvests. Individual values are shown as circles and error bars represent ± SE (standard error) of the mean of four replicates. Data for the genotypes were compared at each harvest with Student's t-test (* $P < 0.05$; ** $P < 0.01$).

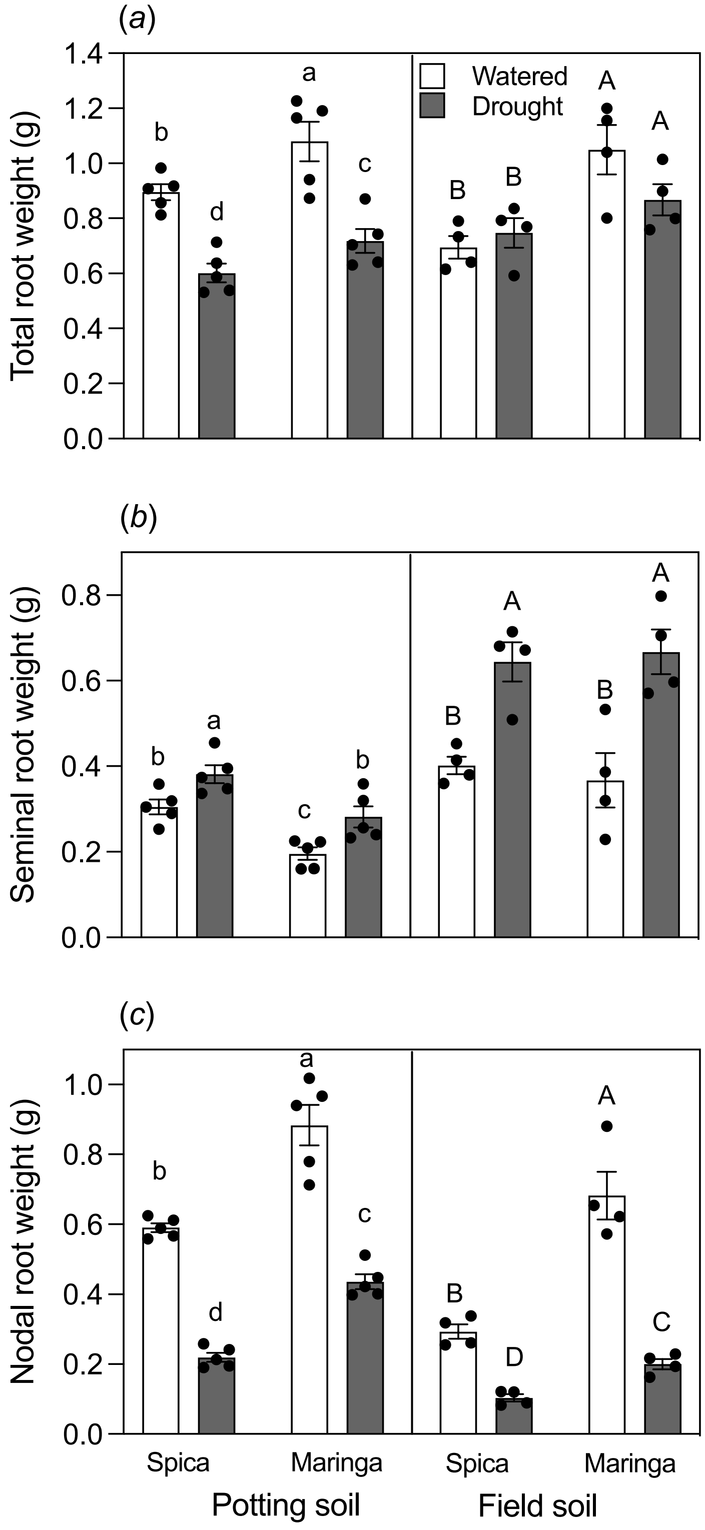

Figure S4. Root weights of Spica and Maringa. Total root weight (a), seminal root weight (b) and nodal root weight (c) of the two genotypes in watered (empty bars) and drought (filled bars) treatments after 28 d growth. Individual values are shown as circles and error bars represent ± SE of the mean for either five replicates (potting soil) or four

replicates (field soil). Data for potting and field soils were analysed separately by two-way ANOVAs. Different lowercase letters reflect significant differences between treatments as determined with the Tukey test at $P < 0.05$ for potting soil whereas different uppercase letters reflect significant differences between treatments at $P < 0.05$ for field soil. The interaction between water and cultivar factors for potting soil was (a) not significant for total root weight, (b) not significant for seminal root weight and (c) not significant for nodal root weight. The interaction between water and cultivar factors for field soil was (a) not significant for total root weight, (b) not significant for seminal root weight and (c) significant at $P < 0.05$ for nodal root weight.

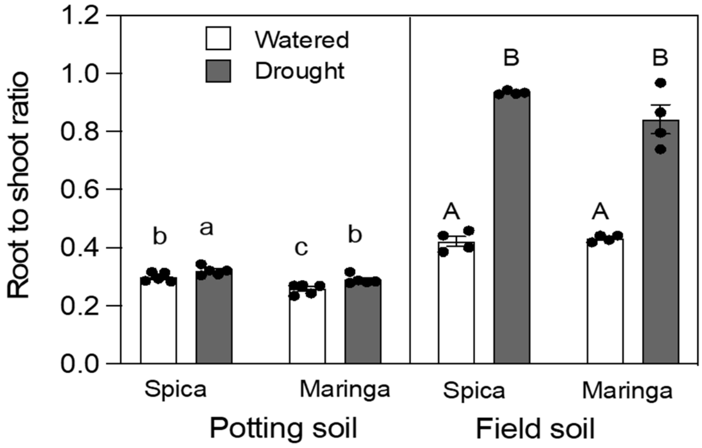

Figure S5. Effect of soil types and drought on root to shoot ratios of Spica and Maringa. The two genotypes were grown in watered (empty bars) and drought (filled bars) treatments for 28 d. Individual values are shown as circles and error bars represent ± SE of the mean for either five replicates (potting soil) or four replicates (field soil). Data for potting and field soils were analysed separately by two-way ANOVAs. Different lowercase letters reflect significant differences between treatments at $P < 0.05$ for potting soil whereas different uppercase letters reflect significant differences between treatments as determined with the Tukey test at $P < 0.05$ for field soil. The interaction between water and cultivar factors was not significant for both potting and field soils.

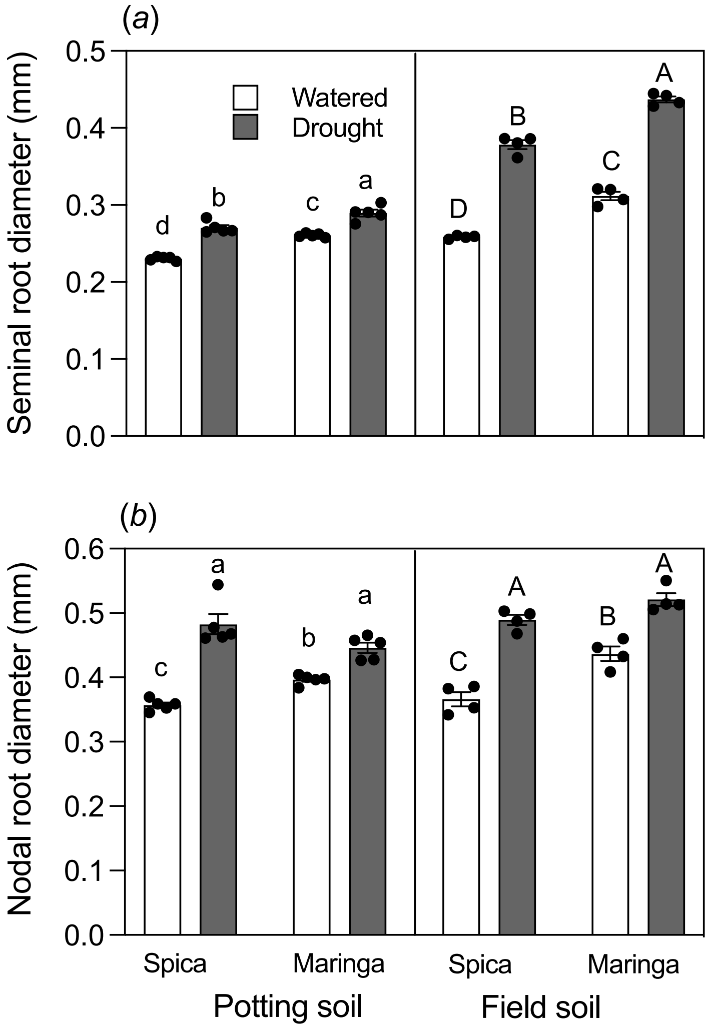

Figure S6. Effects of soil type and water treatment on the average root diameters. Average root diameters of seminal roots (a) and nodal roots (b) of the two genotypes in watered (empty bars) and drought (filled bars) treatments after 28 d growth. Individual values are shown as circles and error bars represent the SE of the mean for either five replicates (potting soil) or four replicates (field soil). Data for potting and field soils were analysed separately by two-way ANOVAs. Different lowercase letters reflect significant differences between treatments at $P < 0.05$ for potting soil whereas different uppercase letters reflect significant differences between treatments as determined with the Tukey test at $P < 0.05$ for field soil. The interaction between water and cultivar factors for potting soil was (a) not significant for seminal root diameter and (b) significant at $P < 0.05$ for nodal root diameter. The interaction between water and

cultivar factors for field soil was (a) not significant for seminal root diameter and (b) not significant for nodal root diameter.

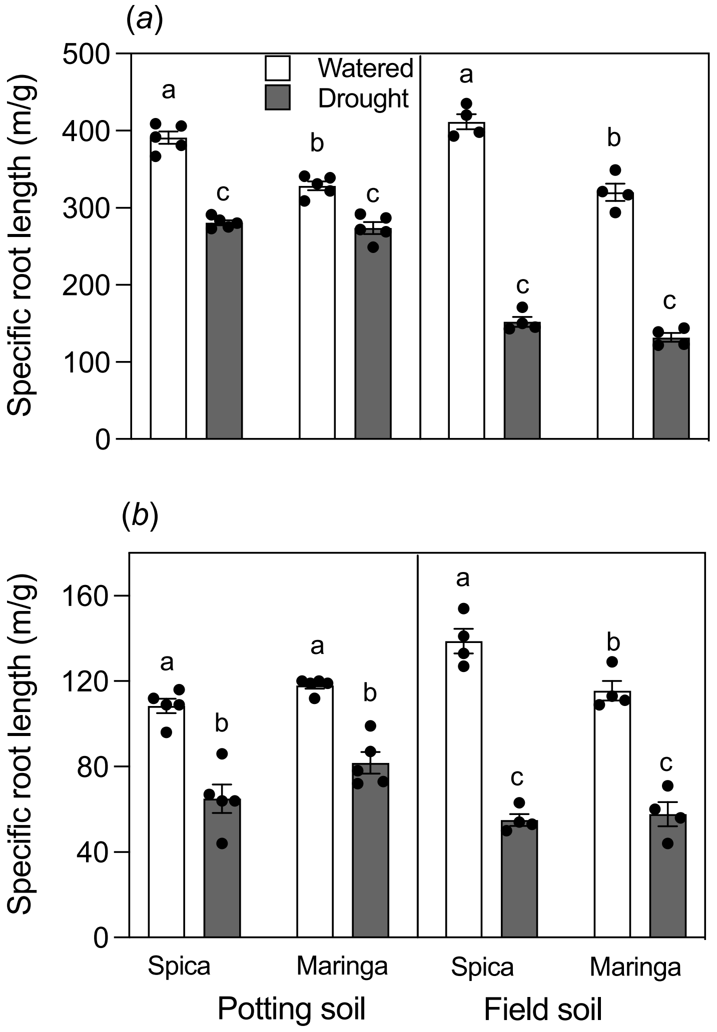

Figure S7. Effects of soil type and water treatment on specific root length. Specific root length of seminal roots (a) and nodal roots (b) of the two genotypes in watered (empty bars) and drought (filled bars) treatments after 28 d growth. Individual values are shown as circles and error bars represent the SE of the mean for either five replicates (potting soil) or four replicates (field soil). Data for potting and field soils were analysed separately by two-way ANOVAs. Different lowercase letters reflect significant differences between treatments at $P < 0.05$ for potting soil whereas different uppercase letters reflect significant differences between treatments as determined with the Tukey

test at $P < 0.05$ for field soil. The interaction between water and cultivar factors for potting soil was (a) significant at $P < 0.05$ for specific root length of seminal roots and (b) not significant for specific root length of nodal roots. The interaction between water and cultivar factors for field soil was (a) significant at $P < 0.05$ for specific root length of seminal roots and (b) significant at $P < 0.05$ for specific root length of nodal roots.

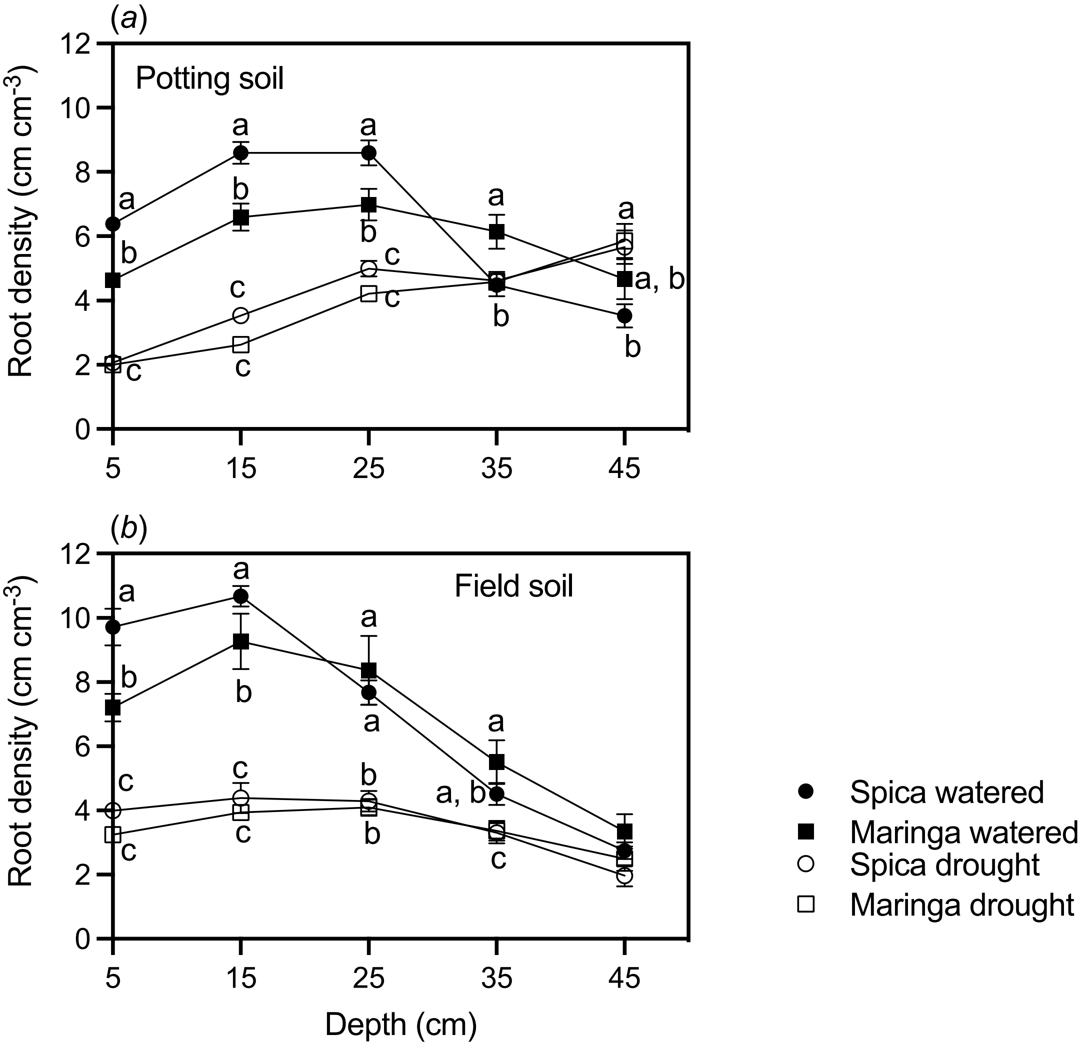

Figure S8. Root density distribution down soil profiles. The roots of plants grown in the tubes for 28 days were washed out and sectioned into 10 cm segments along the whole root system starting from the base. The root segments were then scanned to determine total root length of plants grown in potting soil (a) and field soil (b). The depth designations denote the average depth of each segment such that, for example, the 0–

10 cm segment is shown as having a depth of 5 cm. Watered treatments of Spica (circles) and Maringa (squares) are represented by filled symbols whereas drought treatments are represented by empty symbols. Data show means (n = 5 for potting soil; n = 4 for field soil) and error bars denote the SE. Error bars are obscured if smaller than the symbols. Data at each depth were analysed separately by 2-way ANOVA with different letters indicating significant differences ($P < 0.05$). For potting soil (a) rooting density and cultivar had a significant interaction at depths of 5 and 35 cm otherwise did not interact at other depths. For field soil (a) rooting density and cultivar had a significant interaction at a depth of 5 cm otherwise did not interact at other depths.
